# Evaluation of Compliance in Control and Prevention Study of Vancomycin Resistant Enterococcus Outbreak

**DOI:** 10.1155/2013/252469

**Published:** 2013-02-06

**Authors:** Vuslat Kecik Bosnak, Mustafa Namiduru, Ilkay Karaoglan, Ayse Ozlem Mete

**Affiliations:** Department of Infectious Diseases and Clinical Microbiology, Faculty of Medicine, Gaziantep University, 27310 Gaziantep, Turkey

## Abstract

*Objective*. Vancomycin resistant enterococci (VRE) colonization and the spread decrease with compliance and isolation guidelines. For the isolation methods to succeed, compliance with the isolation guidelines needs to be overseen. In this study, VRE outbreak among the Pediatric Ward patients, the preventative efforts to control this outbreak, and the impact of scoring tables used in controlling this outbreak on the success are explained. *Design*. Rectal swab cultures were taken from patients who were admitted to the Ward between October and December 2010 due to diagnoses of VRE and urinary tract infection. *Results*. VRE were isolated in the rectal swab samples of 34 patients. VRE infection findings were clinically detected in two of the cases with VRE isolation. Two isolations of VRE were detected on cultures from patient room door handle surface and the telephone in the room for common use. Close contact isolation was achieved and barrier precautions were taken for all cases, as soon as the detections were made. A scoring system was developed to evaluate the feasibility of and the compliance with the precautions to be taken. *Conclusions*. With the method presented in this study, the outbreak at our hospital was under control within two months.

## 1. Introduction

Vancomycin resistant enterococci (VRE) are microorganisms that cause epidemics in critically ill patients. Vancomycin resistance in enterococci was first displayed in 1986 [1]. Next, significant increases in the rates of nosocomial infections due to VRE were observed around the world [2, 3]. 

In control and prevention of VRE infections, precautions are taken based on the recommendations of Hospital Infection Control Practices Advisory Committee (HICPAC) that function under Centers for Disease Control and Prevention (CDC) [4]. With these precautions, VRE colonization and spread are decreased. For the isolation methods to succeed, compliance with the isolation guidelines needs to be overseen as well. In this study, VRE colonization that occurs after diagnosis of VRE affected urinary tract infections among the Pediatric Ward patients; the preventative efforts to control this colonization and the impact of scoring tables used in controlling this study on the success are explained.

## 2. Materials and Methods

### 2.1. Setting and Definition of the Cases

Gaziantep University Medical School Hospital is a 930-bed capacity hospital in the Southeast Anatolian Region of Turkey, serving outpatients and inpatients. Pediatric clinic is a 52-bed capacity clinic, where pediatric patient followup and treatments are performed. The Pediatric Ward is spread over two corridors of single and double rooms. The number of Enterococcus strains followed up on in this clinic since January 2010 were detected. In early October, Hospital Infection Control Committee (HICC) was informed that two pediatric patients were diagnosed with urinary tract infection caused by VRE. Next, rectal swab cultures of all inpatient patients between October and December 2010 were obtained. There were 535 inpatients during this time period. The rectal swab samples were taken using Stuart transport medium. Patients with no clinical findings were classified as colonization. 34 patients (20 males, 14 females) with detected VRE colonization were included in the study.

 Information on patients' age, gender, antibiotic use, catheter use, underlying medical illness, primary illness, duration of hospitalization before VRE isolation, and duration of total hospitalization were obtained.

### 2.2. Description of the Outbreak

In this study, two cases with VRE caused urinary tract infection (UTI) diagnosis were defined as index cases. Of these patients, clinical and laboratory findings supported the diagnosis of UTI. These were patients undergoing oncological treatment and long-term antibiotic therapy. Therefore these patients did not treated as outpatient and discharged home. 

 Rectal swab cultures of all inpatient patients were taken throughout the study. Upon the 34 positive outcomes of the patient rectal swab cultures, HICC quickly achieved close contact isolation of patients with VRE colonization. Close contact isolation boards were hung on the patient rooms. Additionally, different surface swab cultures were obtained including the surfaces of the nursing desk, computer keyboard, scale, patient room environment, door handle, patient etagere, and patient bed. 

### 2.3. Laboratory Methods

Colonies isolated in the media were put into identification processes. With strains having negative reactions in catalase tests, black colored *Enterococcus* strains reproduced in the bile-esculin-azide medium under catalase negative 6.5% NaCl conditions were cultivated on the surface of the Mueller-Hinton agar (Oxoid) plates as in the 0.5 McFarland suspension setting standard Kirby-Bauer disk diffusion method for antibiogram.

Antibiotic discs were placed to investigate the susceptibility of the strains. Vancomycin and teicoplanin MIC values were detected with the E test method (AB Biodisc). For *Enterococcus faecium* and *E. faecalis*, vancomycin MIC ≤ 4 *μ*g/mL value was classified as susceptible; MIC = 8–16 *μ*g/mL value was classified as moderately susceptible, and MIC ≥ 32 *μ*g/mL value was classified as resistant [5]. Patients were followed up via weekly rectal swab cultures with regard to VRE colonization. 

### 2.4. Cohorting of VRE Carriers

Patients with VRE infection and colonization diagnoses were put under close contact isolation. The single and double rooms in one of the two corridors of the Pediatric Ward were used for VRE positive patients, while the other corridor was spared as the clean corridor for the patients with negative rectal swab cultures. The common kitchen and the children's playroom for the patient relatives on the Pediatric Ward were temporarily closed, as they could be a source of transmission. Health services personnel and the nurses serving these patients were cohorted. A warning system was created in the hospital computer information system, to make the VRE infected and/or colonized patient information available.

### 2.5. Antibiotic Control Policy

An Infectious diseases specialist at our Hospital makes approvals of antibiotic groups including vancomycin and 3rd generation cephalosporins. This specialist makes advice on antimicrobial prescription to the pediatricians.

Trainings on use of these antibiotics on appropriate indications were offered and antibiotic approvals were closely tracked as VRE positive patients.

### 2.6. Intensified Control Measures Implemented

In order to control the outbreak, a current condition analysis was made by recording the number of inpatients, patients infected with VRE, and patients colonized by VRE. All patients were placed under close contact isolation. A scoring system was developed to evaluate the feasibility of and the compliance with the precautions to be taken (Table 1). This scoring system was based on a form developed according to suggestions made by HICPAC from CDC on how to control the spread of VRE [4]. This form was named “Scoring Form for Fight Against VRE” and consisted of 6 items and a scoring table. Pediatric clinic nurses and HICC nurses made daily tracking of the scoring form. This scoring table consisted of 12 main titles of placing the patient in the room, use of gloves and hand disinfection, the use of gowns, medical devices, surveillance monitoring, physician visit/patient visit/attendant, transportation of patients and helping them leave the room, patient room cleanliness, terminal disinfection/cleaning (after discharge), cleaning and disinfection of the materials used for the VRE positive patient, and the patient's discharge. These titles had subtitles scored out of two each, with a total score of 136. Each item on the task performed was to be scored as yes = 2, no = 0, and sometimes = 1. These forms were scored on daily basis. Additionally, all health personnel working at the Ward were evaluated on their compliance with the scoring table. Daily followups and tracking continued until each item had a full score of 2. Data were recorded on an Excel file. Data analysis was performed using Microsoft Excel 2007 software. The trial was initiated upon approval of local ethical committee.

## 3. Results

There were 535 inpatient patients at the clinic between October and December 2010. Rectal swab samples were obtained from all of the patients. A total of 34 patients, 20 male and 14 female, were detected to have VRE colonization and were included in the study. 

**Figure 1:**
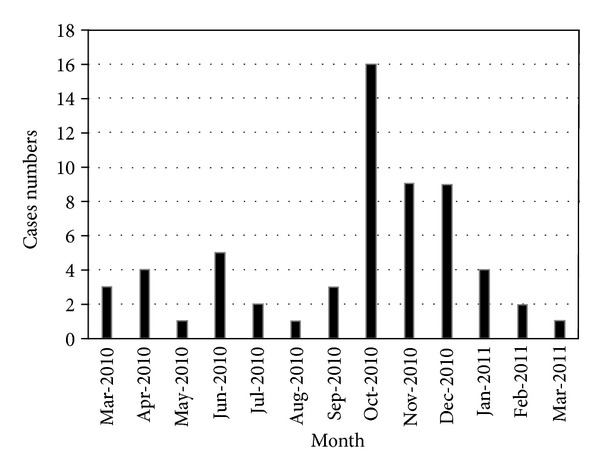
The number of VRE colonized patients between March 2010 and March 2011.

Since March 2010, there were significant increases in the number of VRE colonized patients who were observed during the months of October, November, and December (Figure 1). Monthly VRE colonized patients and the total number of inpatients were summarized in Table 2. Demographic data of VRE colonized patients were summarized in Table 3.

In the rectal swab cultures, there were 21 cases with *Enterococcus faecium, *7 with *E. faecalis, *4 with* E. gallinarum*, and 2 with* E. casseliflavus.* VanA resistance was detected in the isolated VRE strains by Polymerase Chain Reaction (PCR).

Among the samples obtained from the patient rooms, VRE were detected in two sample cultures that were obtained from the door handle surface of the patient room and from the surface of the shared phone in the room.

On the developed form, “Scoring Form for Fight against VRE,” the total score for the first week was 72 out of 136. Significant discrepancies due to implementation mistakes were observed especially in the items pertaining to use of gloves and hand disinfection, the use of gowns, daily patient room cleanliness, terminal disinfection/cleaning (after discharge), and cleaning and disinfection of the materials used for the VRE positive patients. To overcome these discrepancies, HICC offered trainings to health service providers, assistants, faculty members, and patient relatives. In the following weeks, the total scores were added up to 86, 94, 102, 108, 116, 124, 130, and 136; and the colonization was under control within two months.

## 4. Discussion

The first guideline for controlling VRE within hospitals was published in 1994 by HICPAC [4]. HICPAC included suggestions to decrease transmission among the inpatients at the hospitals. These precautions included limiting Vancomycin use, health personnel trainings on hand hygiene, routine scanning for vancomycin resistance among clinical isolates, and putting VRE positive patients under close contact isolation. Society for Healthcare Epidemiology of America (SHEA) emphasized adding routine active surveillance cultures to these suggestions [6].

Despite all these suggestions, studies and the time since, VRE are still an endemic at hospitals with an increasing incidence around the world. Excessive use of antibiotics, use of insensitive methods in stool VRE detection, VRE carriers frequently becoming inpatients at hospitals as transmission sources, and failure to fully comply with infection control methods are among the reasons for this condition [3].

Especially in developing countries with limited resources, factors such as delays in VRE colonization detection, uncontrolled admissions, excessive use of wide spectrum antibiotics, and noncompliance with infection control methods make it difficult to fight against VRE [7]. 

All of these infection control methods, when applied correctly, decrease the frequency of VRE colonization and infection. Still, the most effective method and when it should be used are not clear. Common practice is to isolate contact and to use surveillance cultures in high-risk patients. With the applicable strategies, VRE transmission and carrier patients can be decreased. However, the noncompliance with the infection control methods and hand washing are still viewed as the main issues [3].

There are numerous sources of information and studies in the literature on VRE colonization control. In a study by Eckstein et al. the infection rates were significantly decreased with especially training the personnel who clean the VRE positive patients' rooms [8]. Lai et al.'s study detected a considerable amount of decrease in VRE colonization and infection incidences with the use of waterless alcohol-based hand antiseptics [9]. Moretti et al.'s [7] study reported that a VRE epidemic in a Brazilian hospital was controlled by temporarily not admitting new patients to the clinic involved, shutting down the clinic where the resource patient was staying, obtaining swab cultures for VRE, surveillance monitoring, isolation precautions, and continuous trainings. A study from France, conducted by Aumeran et al. [10], named “Successful control of the VRE Epidemic,” reportedly controlled the epidemic with the close contact precautions involving rectal swab cultures, surveillance monitoring, and hand hygiene. 

Our study implemented a scoring table developed based on the HICPAC suggestions to detect compliance with these suggestions. The noncompliance fields and noncompliant personnel were identified and trainings were offered in light of these findings. While studies conducted are related especially to the hand hygiene suggestions of the infection control methods, we have not come across any studies evaluating and following up compliance with these suggestions. This scoring table is a method that is easy to implement and evaluate the results of as it allows for evaluating the compliance with control methods and directing the trainings accordingly. It also provides a measurable and observable followup opportunity with the gradually increasing compliance and points with the scoring practice. Additionally, it helps identify the non-compliant personnel and the target group for trainings.

The relationship between compliance with control methods and VRE eradication is highly significant in our study. A significant decrease in the VRE frequency was observed beginning especially in the months when compliance with the suggestions was considerably higher. 

Majority of enterococcal infections are thought to result from the patient's endogenous flora for enterococci are an element of the normal gastrointestinal and female genital tract flora. Enterococci are microorganisms that can survive on nonliving surfaces such as patient bed, linen, etagere, wall, and floor for various time periods ranging between 7 weeks and 3 years [11]. VRE colonizes generally on the gastrointestinal tract and on the skin and can survive in the environment. Therefore, the spread of VRE occurs usually through direct contact with colonized or infected individuals, indirect contact with health personnel's hands, or contact with contaminated equipment or surfaces [12]. While environmental cleaning to eliminate environmental contamination is very important in preventing the spread of microorganisms, a study reports that despite frequent room cleanings, nosocomial transmission of VRE was shown to be associated with room contamination [13]. There were VRE contaminations detected on patient room door handle and shared room telephones in the surface cultures obtained in the beginning of our study, which were not found in the repeat environmental cultures obtained after the scoring was implemented and the trainings offered. A decrease in the number of patients with VRE colonization along with the decreased environmental contamination demonstrates the importance of environmental cleanliness. 

Patient-to-patient transmission is also likely to happen via direct or indirect contact with contaminated hands, contaminated surfaces, or medical devices [3]. Therefore it is crucial to have clean surfaces and to form the hand washing habit. Duckro et al. have demonstrated that VRE are transmitted through health personnel's' hands [14]. On the other hand, numerous studies show that hand washing compliance can be very low [3, 15]. With the scoring table used in our study, especially hand washing related discrepancies were identified and the personnel were trained primarily on hand washing. 

Among the vancomycin resistant strains of enterococci, *Enterococcus faecium* and *Enterococcus faecalis* were the most frequently isolated ones [16]. These are usually resistant to VanA gene. The most common type of enterococci in the colonization that occurred in our hospital's Pediatric Ward was *Enterococcus faiecum.*

VRE colonization/infection is observed more frequently in some patient groups and some VRE risk definitions have been attempted at. Long-term antibiotic (vancomycin, cephalosporins, and antianaerop agents) use, presence of neutropenia, prolonged hospital stays, and intensive care unit hospitalizations are identified risk factors. Patients staying at the hematology-oncology department are in the risk group [17]. There was a history of long-term antibiotic use in 20 (58.8%), chronic renal diseases in 7 (20.6%), and long-term catheter use in 4 (11.8%) of the patients in our study. According to the demographic data, 58.8% and 20.6% of the patients had significantly high history of long-term antibiotic use and chronic renal diseases, respectively. 

VRE colonization that occurred after detection of urinary tract infections associated with VRE among the patients staying at the Pediatrics Ward, and to control this colonization, HICC started to take precautions based on the suggestions of HICPAC of CDC on how to control the spread of VRE [4]. These precautions included limiting vancomycin use, health personnel trainings on hand hygiene, routine scanning for vancomycin resistance among clinical isolates, putting VRE positive patients under close contact isolation, and monitoring of rectal surveillance cultures. The most important thing in controlling the epidemic is to comply with the precautions taken under guidance of HICPAC suggestions and to closely track them. Even if the patients are under close contact isolation, mistakes and discrepancies in the applications make the epidemic control more difficult.

## 5. Conclusion

In prevention of hospital-acquired infections, it is important both to take precautions regarding close contact isolation and to follow up on the implementation of these precautions. Different methods can be used for the followup of these methods. With the method presented in this study, the colonization at our hospital was under control within two months. Active cooperation with the clinic is essential in this regard. The colonization can be controlled with early precautions taken on close contact isolation and close tracking of it, along with trainings offered at and active cooperation with the clinic involved. 

## Figures and Tables

**Table 1:** Scoring Form for Fight against VRE.

(i) Placing the patient in the room (10 points total)	Score
(1) Patient colonized or infected with VRE is preferably placed in an isolated room with own toilet and sink. If no isolated room is available, s/he will be placed in a room with other patients colonized with VRE.	
(2) Personnel responsible for the care of the patient(s) colonized/infected with VRE are responsible for care of only this/these patient(s).	
(3) Patient files and charts are outside the patient's room.	
(4) A toilet in the common bathroom is spared for this/these patient(s) and is cleaned with a 500 ppm sodium hypochlorite solution after each use.	
(5) If personnel remove the stool of the patients in rooms with no toilet, the personnel wear a long gown and gloves and wash hands after removal.	

(ii) Use of gloves and hand disinfection (10 points total)	Score

(1) While entering the patient room, clean and nonsterile gloves are worn before any contact with the patient or the surrounding surfaces.	
(2) Gloves are changed after procedures that can cause contamination during patient care (contact with stool, infected wound drainage).	
(3) Gloves are taken off before leaving the patient room.	
(4) After the gloves are removed, hands are washed with an antimicrobial agent (chlorhexidine).	
(5) Hands are cleaned with an alcohol-based hand disinfectant if no visible contamination with bodily fluids such as blood, feces, and so forth has occurred.	

(iii) Use of gowns (4 points total)	Score

(1) Clean, nonsterile gowns are worn before entering the patient room and are taken off before leaving the room.	
(2) If there is a possibility of contact with blood and other bodily fluids, a plastic gown is worn over.	

(iv) Medical devices (6 points total)	Score

(1) All medical devices used are patient specific (stethoscope, sphygmomanometer, thermometers, sliders, etc.).	
(2) If common use is necessary, these devices are cleaned and disinfected before use on other patients.	
(3) Noncritical devices are stored in the patient room in small amounts (e.g., bandages).	

(v) Surveillance monitoring (14 points total)	Score

(1) Rectal swab and stool samples are obtained from all patients in the same unit with the new cases diagnosed with VRE.	
(2) Close contact isolation precautions are attained for cases with VRE detected in culture results.	
(3) Weekly surveillance cultures are obtained from VRE positive patients.	
(4) Close contact isolation is terminated if three consecutive weekly surveillance cultures return negative.	
(5) Isolation termination decisions are made with HICC on patient by patient basis.	
(6) All room surfaces are disinfected upon discharge and environmental cultures are obtained from the room.	
(7) No new patients are placed in these rooms and it is prevented to use these devices on other patients until environmental culture results are obtained.	

(vi) Physician visit/patient visit/attendant (10 points total)	Score

(1) Only personnel on duty work on the isolated grounds outside the visits. Unless necessary, personnel access from other areas is limited.	
(2) Only personnel on duty work on the isolated grounds during night shifts outside the visits. Unless necessary, personnel access from other areas is limited.	
(3) Access to and from the patient(s) room is limited.	
(4) Patient attendants are informed about and trained on the matter.	
(5) Visitors can see the patients within short time upon abiding by the rules.	

(vii) Transportation of patients and helping them leave the room (12 points total)	Score

(1) Patients leave their rooms for as little as possible.	
(2) Patients can leave the room if they do not have a wound or a lesion.	
(3) Patients wash their hands with chlorhexidine before leaving their room and can wander in the corridor or on the balcony.	
(4) If the patient has to leave the room, his/her destination is informed and necessary precautions are taken.	
(5) If the patient is carried with a wheelchair or a stretcher, these tools are covered with a clean cover, which is removed and put into a special laundry bag after use. These tools are thoroughly cleaned with alcohol after use.	
(6) If the patient's general condition is better, he/she is informed about his/her condition and what he/she should do.	

(viii) Cleanliness of the patient room (during stay) (12 points total)	Score

(1) Cleaning equipment (buckets, cloths, mops, mop handles, etc.) for the patient room is specific to the room and cannot be used in other rooms.	
(2) Floors are mopped with detergent water twice and as they get dirty during each shift.	
(3) The mops are disinfected after each use with 1% sodium hypochlorite water.	
(4) The furniture, bedding, serum hangers, patient transfer vehicles, and so forth in the patient room are disinfected with 1% sodium hypochlorite water after cleaning.	
(5) The floor and surfaces are not left wet.	
(6) If the floor or the surfaces become contaminated with organic substances (blood, urine, stool, etc.), it is absorbed fully with a paper towel with gloves on and disinfected with 10% sodium hypochlorite water. It is left as it is for 10 minutes and then recleaned with warm detergent water and dried.	

(ix) Terminal disinfection/cleaning (after patient is discharged) (10 points total)	Score

(1) Room and patient equipments are cleaned first with detergent and warm water.	
(2) Next, they are disinfected with 1% sodium hypochlorite solution.	
(3) All surfaces: walls, bathroom, toilets, doors and door knobs, the whole bed, telephone, television, monitor, call bells, and so forth with possible contamination are cleaned.	
(4) Curtains and all furniture with cloth are changed and washed.	
(5) All surfaces, after wiping with the disinfectant, are rinsed with water and dried.	

(x) Cleaning and disinfection of devices used for patients with VRE (16 points total)	Score

(1) Patient beds are covered with a protector. The protector is cleaned after patient is discharged, and disinfected with a 70% alcohol or 1% sodium hypochlorite solution.	
(2) Patient linens are placed in a waterproof bag with slow movements to avoid spread of pathogens into the air, sealed, and sent to the laundry.	
(3) If the outer surface of the laundry bag is contaminated, a second bag is used.	
(4) Slides and ducks are soaked in 1% hypochlorite solution for 20 minutes and drained upon discharge.	
(5) Thermometers are disinfected in a 20% alcohol solution.	
(6) Stethoscope is wiped with a 70% alcohol solution upon discharge.	
(7) Sphygmomanometer's metal and inner rubber parts are removed and wiped with a 70% alcohol solution. Its case is soaked either in a 10% lanyard or 1-2% sodium hypochlorite water and then rinsed.	
(8) All of the materials from VRE patients are medical waste (e.g., food bag, water bottle, coke can, etc.). All of them are disposed into the red bin as long as the patient is isolated.	

(xi) Patient discharge (12 points total)	Score

(1) Colonized or infected patients' data are systematically recorded in his/her patient file.	
(2) The patient is discharged as soon as possible if his/her conditions allow.	
(3) If the patient is rehospitalized at the same hospital, s/he will be under close contact isolation until a negative surveillance VRE culture is obtained.	
(4) While transferring a patient to another health institution, the head offices are informed about the matter.	
(5) Ambulance used on patient transfer is warned to take the necessary precautions.	
(6) If a patient is transferred from another hospital and has the risk factors mentioned above s/he will be under close contact isolation until a negative surveillance VRE culture is obtained.	

(xii) Compliance with action plan (20 points total)	Score

(1) Faculty members	
(2) Residents	
(3) Interns	
(4) Other consultants	
(5) 4-5 year student	
(6) Nurses	
(7) Cleaning personnel	
(8) Other personnel	
(9) Patient attendants	
(10) Patients	

Total	

Fill out the empty box next to the performed task as yes = 2, no = 0, and sometimes = 1.	

**Table 2:** Monthly VRE-colonized and the total number of inpatients.

Date	VRE colonized patients	The number of inpatients
Mar-2010	3	175
Apr-2010	4	195
May-2010	1	176
Jun-2010	5	199
Jul-2010	2	176
Aug-2010	1	202
Sep-2010	3	199
Oct-2010	16	162
Nov-2010	9	164
Dec-2010	9	209
Jan-2011	4	196
Feb-2011	2	149
Mar-2011	1	192

**Table 3:** Demographic data of VRE colonized patients, *n* (%).

Gender (M/F)	20/14
Age	4.05 ± 4.91 years
Duration of hospitalization before VRE isolation (mean ± SD)	9 ± 10.9 days
Long-term antibiotic use	20 (58.8)
Long-term catheter use	4 (11.8)
Underlying medical illness	
Chronic renal diseases	7 (20.6)
Neurometabolic diseases	5 (14.7)
Oncological diseases	4 (11.8)
Chronic respiratory diseases	2 (5.8)
Cerebrovascular diseases	1 (2.9)
Primary illness	
Pneumonia	9 (26.5)
Fever	6 (17.6)
Cardiac failure	4 (11.8)
Peritonitis	4 (11.8)
Urinary tract infections	2 (5.9)
Others	9 (26.5)
Duration of total hospitalization (mean ± SD)	14 ± 13.86 days
Enterococcal strain species in positive cultures	
*E. faecium *	21 (61.7)
*E. faecalis *	7 (20.5)
*E. gallinarum *	4 (11.7)
*E. casseliflavus *	2 (5.8)
